# Comparison of three methods for measuring C0-1 angles and C0-2 angles

**DOI:** 10.1186/s12891-023-06402-0

**Published:** 2023-04-18

**Authors:** Shicai Xu, Fei Ma, Chao Tang, Yehui Liao, Qiang Tang, Shiyu Chen, Qing Wang, Dejun Zhong

**Affiliations:** Department of Orthopaedics, The Affiliated Hospital of Southwest Medical University, No.25 Taipingjie, Lu Zhou, Sichuan, 646000 PR China

**Keywords:** Cervical sagittal parameters, Measurement, Reliability, Correlation

## Abstract

**Background:**

The mutual compensatory relationship between the upper cervical sagittal alignment and the lower cervical sagittal alignment has been repeatedly reported. However, the evaluation of the upper cervical sagittal parameters are varied in previous studies. This retrospective study was performed to compare three methods for measuring the upper cervical sagittal parameters.

**Methods:**

A total of 263 individuals with standing neutral lateral cervical radiographs were included in this study. The Frankfort horizontal line (FHL), foramen magnum line (FML), and McGregor line (ML) were separately used as the reference lines for measuring the C0-1 angle and C0-2 angle. Intraclass correlation (ICC) values were used to compare the consistency and repeatability of the three methods. Pearson’s correlation analysis was used to analyze the correlation between the sagittal parameters of the upper and lower cervical spine.

**Results:**

The interobserver and intraobserver ICC values obtained from using the ML to measure the C0-1 angle and C0-2 angle were both higher than those obtained from using the FML or FHL. The C0-1 angle and C0-2 angle measured by the three methods were negatively correlated with the C2-7 angle. The upper sagittal parameters measured by the FHL were the most correlated with the C2-7 angle. The correlation between the C0-1 angle measured by the three methods and the C0-2 angle measured with the FHL or ML and the C2-7 angle increased with aging.

**Conclusion:**

Use of the ML to measure the C0-1 angle and C0-2 angle has higher reliability. Use of the FHL to measure the sagittal alignment of the upper cervical spine is more suitable for evaluating the compensation mechanism between the upper and the lower cervical spine.

## Introduction

Over the past few decades, increasing attention has been given to the role of the mutual compensation between the upper and lower cervical spinal alignment in maintaining the sagittal balance of the cervical spine and horizontal gaze [1, 2]. The kyphosis of the upper cervical spine caused by different craniovertebral junction pathologies or surgery of the upper cervical spine recruits a compensatory mechanism to increase lower cervical lordosis [3, 4]. Kyphosis of the lower cervical spine is compensated for by the increase in upper cervical lordosis. To evaluate the parameters of cervical sagittal alignment and analyze the compensation mechanism between upper and lower cervical sagittal alignment in the normal population and in patients with cervical spine disorders, many different evaluation parameters of cervical sagittal alignment have been proposed [5]. The C0-1 angle, C0-2 angle, and C1-2 angle were the most commonly used parameters to evaluate the sagittal alignment of the upper cervical spine. The correlation between these three parameters and the lower cervical sagittal alignment (C2-7 angle) has been repeatedly reported in normal people and patients with upper or lower cervical spine diseases [2, 5, 6].

In past studies, different reference lines on the skull were used to measure the C0-1 angle and C0-2 angle [5, 7–9]. The reference line on C1 was also different when measuring the C0-1 angle and C1-2 angle [8, 10, 11]. The Frankfort horizontal line (FHL), foramen magnum line (FML), and McGregor line (ML) were previously commonly used as the reference lines while measuring the C0-1 angle and C0-2 angle [7–9]. In the evaluation of upper cervical sagittal alignment, it is more important to choose a measurement method with high consistency and repeatability. However, no study has compared the consistency and repeatability of these methods. Therefore, the purpose of this study was to compare the reliability of using FHL, FML, and ML to measure C0-1angle and C0-2 angle. We analyzed the correlation between measurement results of the three methods and sagittal alignment of the lower cervical spine, which is expected to facilitate the selection of a more ideal measurement method for upper cervical sagittal alignment.

In addition, cervical sagittal radiographic alignment changes with age and sex in asymptomatic adults [2, 12, 13]. However, few studies have specifically evaluated changes in correlations of upper and lower cervical sagittal alignment in various age groups. Therefore, we also explored the correlations of sagittal parameters of the upper and lower cervical spine in different age groups and analyzed the effect of sex on correlations of upper and lower cervical sagittal parameters.

## Materials and methods

### Clinical data

#### Ethical approval

was obtained before this retrospective study was initiated (number: KY2022140). All of the methods were performed in accordance with the guidelines and regulations of the ethics review board. The inclusion criteria for enrollment were an asymptomatic status and standing neutral lateral cervical radiographs taken from June 1, 2019, to June 30, 2021. The exclusion criteria were as follows: (1) history of spinal trauma or other diseases, (2) previous spinal surgery, (3) lower extremity disease or surgery, and (4) inferior aspect of C7 revealed on lateral cervical radiographs. Eventually, 263 patients with standing neutral lateral cervical radiographs were included in this study. Among the 263 patients, the average age was 50.4 ± 16.2, and 130 patients were female. Patients were divided into the following age groups: <35 years, 35–49 years, 50–64 years, and > 65 years.

### Measurement of sagittal parameters

**Fig. 1:**
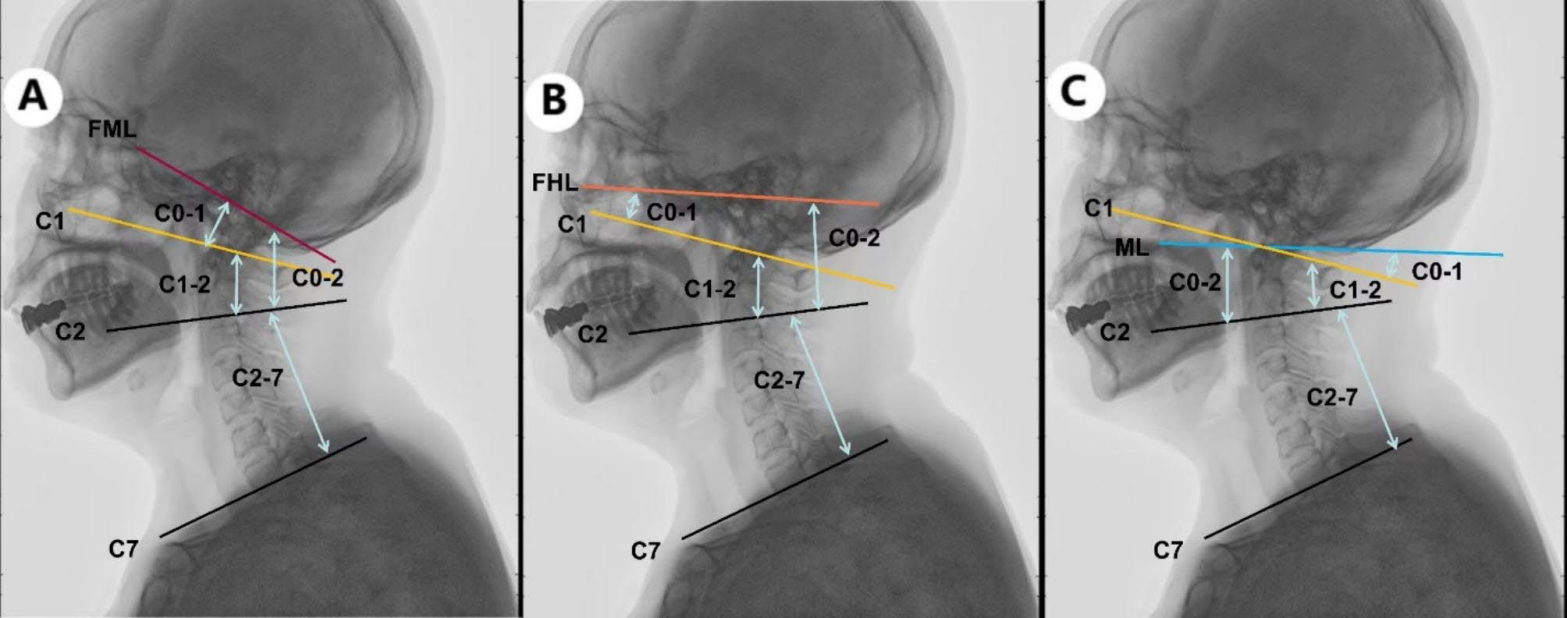
Illustrative explanation of sagittal cervical parameters

The sagittal cervical parameters were measured on lateral cervical radiographs (Fig. 1), as follows: C0-1 angle: (1) angle between the FHL and the superior aspect of the atlas; (2) angle between the FML and the superior aspect of the atlas; (3) angle between the ML and the superior aspect of the atlas. C0-2 angle: (1) angle between the FHL and the inferior aspect of the axis; (2) angle between the FML and the inferior aspect of the axis; (3) angle between the ML and the inferior aspect of the axis. The C1-2 angle is the angle between the superior aspect of the atlas and the inferior aspect of the axis (the inferior aspect of the atlas is difficult to accurately measure because the tail of the C1 screw will obscure the middle-lower part of the posterior arch of the atlas after atlantoaxial fixation surgery). The C2-7 angle is the angle between the inferior aspects of the axis and C7. Measurement of these radiographic parameters was independently and blindly completed on the X-ray machine advantage workstation (ADW) by two spinal surgeons (FM and SCX.) to analyze the interobserver reliability. These two examiners measured the parameter in two sessions at weekly intervals to analyze the intraobserver reliability. The second of the measurements from MF was used to analyze the correlation between the upper and lower cervical spine.

### Statistical analysis

The statistical evaluation was performed using SPSS 26.0 (IBM Corp, Armonk, NY, USA). Quantitative data are presented as the mean ± standard deviation (SD). The independent t test was applied to compare the data between the male group and female group. The normal distribution of parameters was assessed followed by analysis of variance for comparison of variance between age groups, and least significant difference (LSD) post hoc analysis was used for all individual group comparisons. The repeatability and consistency of the measurement method were evaluated using intra-class correlation coefficient (ICC) values(ICCs were defined as follows: 0 to 0.2 slight agreement, 0.21 to 0.4 fair agreement, 0.41 to 0.6 moderate agreement, 0.61 to 0.8 substantial agreement, and 0.81 to 1.0 excellent agreement.). Pearson’s correlations were calculated to examine the relationship between upper and lower cervical sagittal alignment. A p value of less than 0.05 was considered statistically significant.

## Results

### Intrarater and Interrater Reliability

Using the ML as a reference line to measure the C0-1 angle and C0-2 angle yielded higher ICC values of interobserver and intraobserver reliabilities than did corresponding use of the FML or FHL. The ICC values of interobserver and intraobserver reliability of the new method for measuring the C1-2 angle were 0.934 and 0.940, respectively, P < 0.001 (Table 1).

**Table 1:** The results of interobserver and intraobserver reliability of upper cervical spinesagittal parameters

	C0-1 A			C0-2 A			C1-2 A
	FML	FHL	ML	FML	FHL	ML	
	ICC	ICC	ICC	ICC	ICC	ICC	ICC
Reliability of interobserve							
The first measurement	0.830^**^	0.935^**^	0.959^**^	0.880^**^	0.926^**^	0.959^**^	0.918^**^
The second measurement	0.842^**^	0.929^**^	0.969^**^	0.873^**^	0.941^**^	0.978^**^	0.950^**^
Overall	0.836^**^	0.932^**^	0.964^**^	0.876^**^	0.933^**^	0.968^**^	0.934^**^
Reliability of intraobserver							
The first measurement	0.850^**^	0.933^**^	0.964^**^	0.881^**^	0.937^**^	0.974^**^	0.929^**^
The second measurement	0.859^**^	0.950^**^	0.967^**^	0.882^**^	0.949^**^	0.976^**^	0.952^**^
Overall	0.854^**^	0.941^**^	0.965^**^	0.881^**^	0.943^**^	0.975^**^	0.940^**^

**the diference was statistically signifcant(P<0.001)

### Radiographic Parameters

The values of radiographic parameters are shown in Table 2. The C2-7 angle demonstrated significant increases with aging. C2-7 lordosis was significantly greater in patients ≥ 65 years than in those < 50 years (P < 0.05). Patients who were < 35 years of age were also found to be significantly less lordotic than those > 50 years of age (P < 0.01). The C0-1 angle, C0-2 angle and C1-2 angle were not significantly different among age groups (P>0.05).The mean C0-1 angle(measured by using the FHL and ML as reference lines)and C1-2 angle in male group were lower than those in female group (P < 0.05). The C0-1 angle measured by using the FML as a reference line, the C0-2 angle measured by the three methods and the C2-7 angle were not significantly different between the sex groups.

**Table 2:** Cervical Sagittal Radiographic Parameters

	C0-1 A			C0-2 A			C1-2 A	C2-7 A
Gender(n)	FML	FHL	ML	FML	FHL	ML		
Male(133)	6.8±6.9	-13.1±8.7	-9.7±7.7	28.8±8.6	12.2±5.7	16.2±6.3	26.7±5.4	13.8±11.0
Female(130)	7.1±5.3	-16.1±6.6	-12.2±5.4	29.5±8.1	12.7±6.4	15.9±6.5	28.1±5.2	12.0±10.2
	P=0.697	P=0.002^*^	P=0.002^*^	P=0.493	P=0.478	P=0.722	P=0.036^*^	P=0.185
Age group, years(n)								
20-34(47)	5.1±7.6	-13.3±10.4	-9.6±8.7	30.4±8.1	13.1±6.6	16.8±6.4	28.1±5.1	9.0±10.4
35-49(81)	6.8±6.2	-13.6±8.7	-10.4±7.4	28.0±8.0	12.8±6.5	16.0±6.8	27.0±6.1	11.8±10.8
50-64(77)	7.3±4.8	-15.6±4.9	-12.1±4.7	29.7±7.2	11.9±5.3	15.6±5.7	27.3±4.6	14.4±10.3
≥65(58)	8.1±6.4	-15.7±7.1	-11.1±6.2	29.0±10.3	12.3±5.9	16.1±6.6	27.5±5.2	15.8±10.2
	P=0.094	P=0.173	P=0.215	P=0.412	P=0.663	P=0.795	P=0.705	P=0.005^*^
Total(263)	6.9±6.2	-14.6±7.8	-10.9±6.7	29.2±8.4	12.5±6.0	16.0±6.4	27.4±5.3	12.9±10.7

*Statistically significant

### Correlation analysis and Linear Regression Analysis

**Fig. 2:**
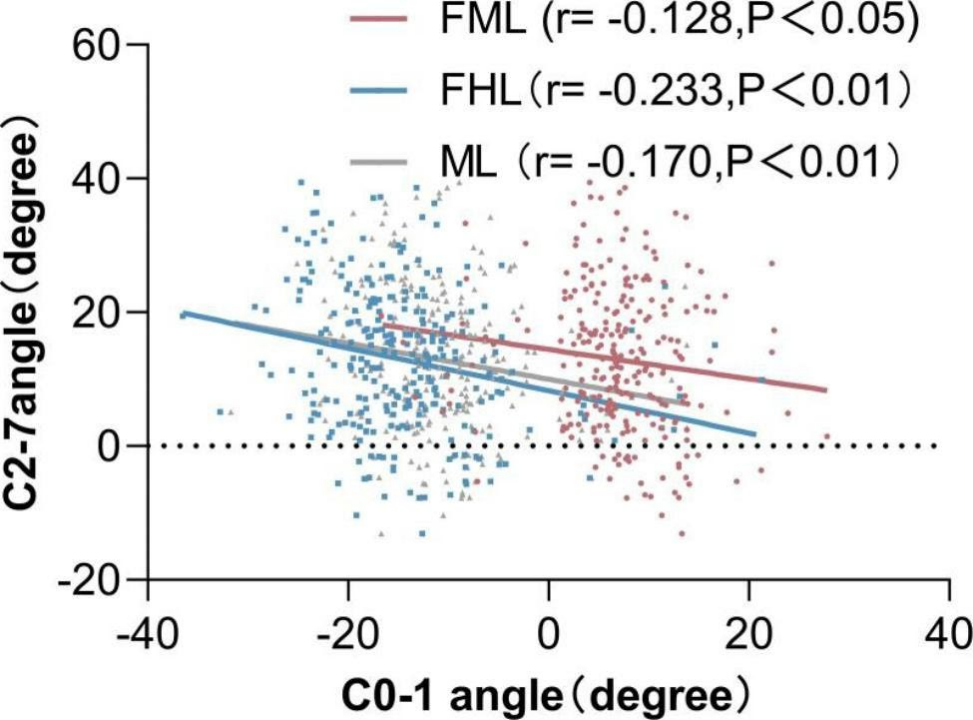
Correlation between C2-7 angle and C0-1 angle measured by three methods

**Fig. 3:**
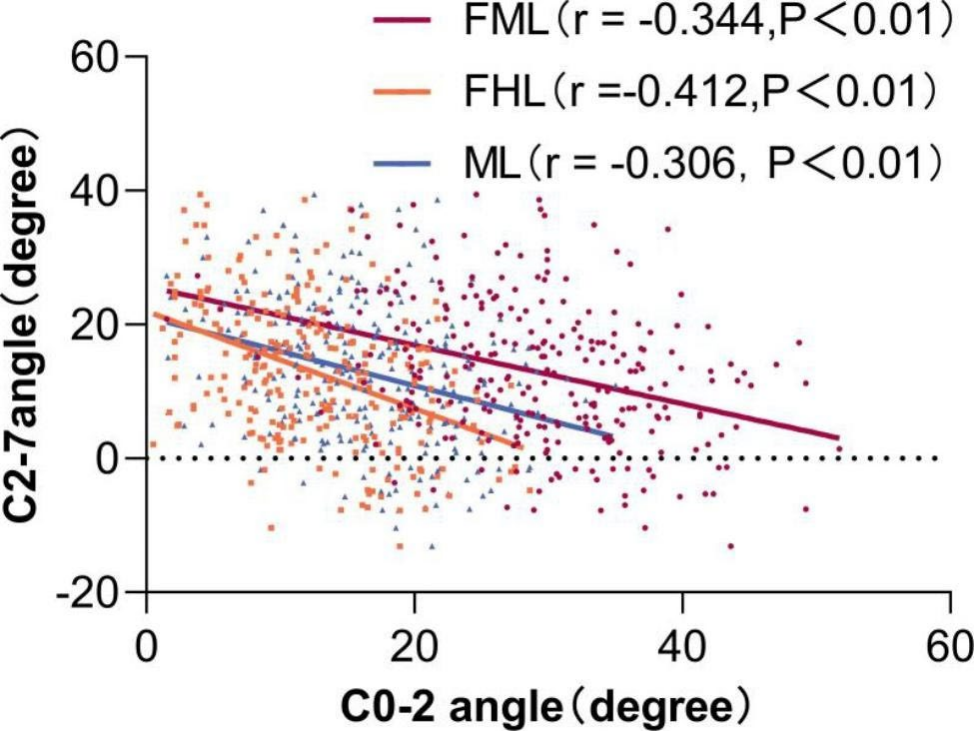
Correlation between C2-7 angle and C0-2 angle measured by three methods

**Fig. 4:**
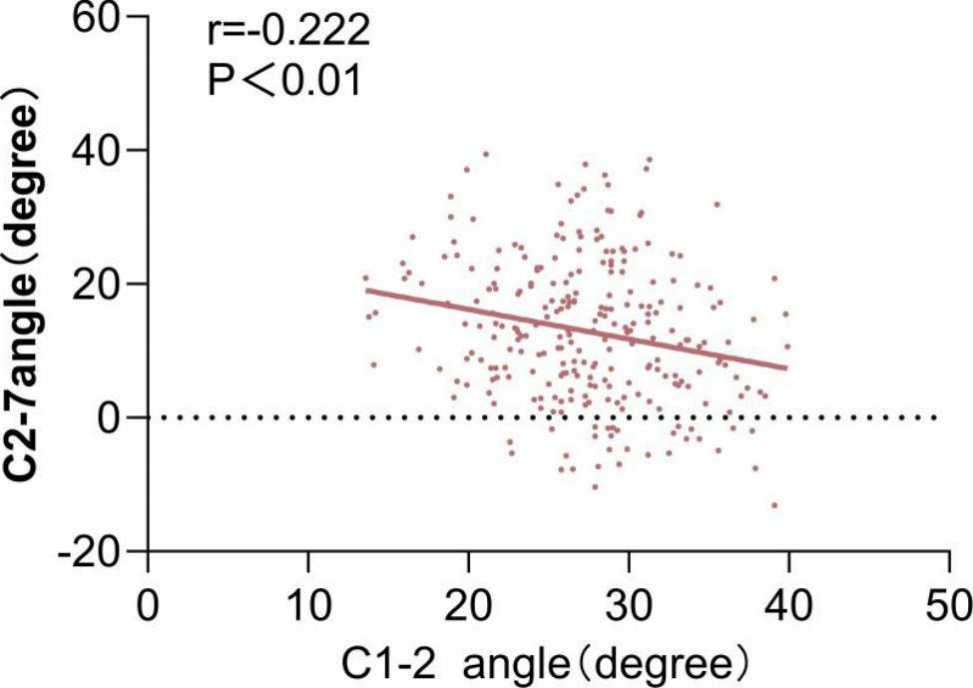
Correlation between C2-7 angle and C1-2 angle

The correlations between the upper and lower sagittal parameters are shown in Table 3. There were significantly negative correlations between the C0-1 angles and C0-2 angles measured by the three methods,and C1-2 angles and the C2-7 angle.Using the FHL as a reference line to measure the C0-1 angle and C0-2 angle yielded higher Pearson correlation coefficient values than using the FML or ML. The results of linear regression analysis are shown in Figs. 2, 3 and 4. The Pearson correlation coefficient values between the C0-1 angles measured by the three methods and the C2-7 angle increased with aging. The correlations between the C0-2 angles measured by using the FHL and ML as reference lines and the C2-7 angle increased with aging. In all age groups, using the FHL as a reference line to measure the C0-1 angle and C0-2 angle also yielded higher Pearson correlation coefficient values than using the FML or ML. The correlation coefficient between the C0-2 angle using the FML as the reference line and the C2-7 angle in patients ≥ 65 years old group was stronger than that of the other age groups.The correlations between the C0-1, C0-2 and C1-2 angles and the C2-7 angle were stronger in females than in males.

**Table 3:** Correlation between the upper and lower cervical sagittal parameters

	C0-1 A			C0-2 A			C1-2 A
Gender(n)	FML	FHL	ML	FML	FHL	ML	
	r	r	r	r	r	r	r
Male(133)	-0.111	-0.166	-0.146	-0.323^**^	-0.366^**^	-0.281^**^	-0.195^*^
Female(130)	-0.149	-0.386^**^	0.261^**^	-0.364^**^	-0.457^**^	-0.340^**^	-0.234^**^
**Age group, years(n)**							
20-34(47)	-0.157	-0.154	-0.021	-0.391^**^	-0.327^**^	-0.184	-0.211
35-49(81)	-0.067	-0.196	-0.118	-0.243^*^	-0.348^**^	-0.277^*^	-0.219^*^
50-64(77)	-0.193	-0.246^*^	-0.219	-0.360^**^	-0.451^**^	-0.335^**^	-0.179
≥65(58)	-0.306^*^	-0.312^**^	-0.306^*^	-0.449^**^	-0.539^**^	-0.409^**^	-0.291^*^
Total	-0.128^*^	-0.233^**^	-0.170^**^	-0.344^**^	-0.412^**^	-0.306^**^	-0.222^**^

*Statistically significant (P<0.05) **Statistically significant (P<0.01)

## Discussion

Previous studies have measured the C0-1 angle, C0-2 angle, and C1-2 angle in normal or asymptomatic people to obtain the normal range of these parameters and guide the selection of the C0-2 angle and C1-2 angle in occipitocervical fixation and atlantoaxial fixation to avoid abnormal changes in the lower cervical curvature or other postoperative complications caused by inappropriate C0-2 and C1-2 angles [1, 14, 15]. These parameters have also been used to analyze and study the correlation of sagittal alignment and the mechanism of mutual compensation of the upper and lower cervical spine. It has been reported that in occipitocervical fusion, a C0-2 angle that is too small may lead to pharyngeal stenosis and dysphagia, while a C2-7 angle will result in compensatory enlargement [16]. Tang et al. also reported that a small C0-2 angle is associated with postoperative implant failure [15]. If the C0-2 angle is too large, the lower cervical spine may have a compensatory decrease in lordosis or even kyphosis [14]. A retrospective study by Yoshimoto et al. found that surgical fixation of the atlantoaxial joint in a hyperlordotic position will lead the lower cervical spine into a kyphotic sagittal alignment after C1-2 fixation and fusion [11]. Biomechanical studies have also shown that C1-2 angle fixation in a neutral position adds 10° to increase intradiscal pressure in the lower cervical spine, which may lead to accelerated degeneration of the lower cervical spine [17]. However, there was no significant difference in the intradiscal pressure of the lower cervical spine between fixation in a neutral position minus 10° and fixation in a neutral position. Therefore, it is recommended to fix the C1-2 angle in a neutral position or a relatively smaller angle. It has been proven that the range of motion of C0-1 in the sagittal plane plays a vital role in compensation after atlantoaxial fixation and cervical kyphosis surgery. Kim et al. revealed that a small ROM at the C0-1 segment is a main risk factor for lower cervical kyphotic change after C1-2 fixation [6]. Miyamoto et al.‘s study demonstrated that the C0-1 angle is more important than the C1-2 angle in the compensatory mechanism for kyphotic deformities at the lower cervical spine [8].

At present, the methods of measuring the C0-1 angle, C0-2 angle, and C1-2 angle are multitudinous in different studies [7–9]. This is mainly shown in the different reference lines selected on the skull when measuring the C0-1 angle and C0-2 angle and the different reference lines selected on C1 when measuring the C0-1 angle and C1-2 angle. Matsunaga et al. measured the C0-2 angle formed by the McGregor line and the inferior surface of the axis in 240 healthy volunteers, which is also the most commonly used measurement method for the C0-2 angle [1]. Guo et al. also used the McGregor line as a reference line to measure the C0-1 angle and C0-2 angle in asymptomatic volunteers. Their correlation analysis showed that the C0-1 angle and C0-2 angle were negatively correlated with the C2-7 angle [18]. A negative correlation was also found between the C0-2 angle and the C2-7 angle when using the MacRae line as the reference line to measure the C0-1 angle and the C0-2 angle in patients without spinal deformities [8]. In addition, Liu et al. used the Frankfort horizontal line as the reference line on the skull and defined the angle formed by the Frankfort horizontal line and the inferior surface of the axis as the Frankfort axial angle [7]. This measurement method conducted by Liu et al. is similar to using the McGregor line or MacRae line as the reference line, which indicates an angle between the reference lines selected on the skull and the C2 vertebral body; therefore, we can also regard the Frankfort axial angle as the C0-2 angle. The present studies showed that the McGregor line, MacRae line, or Frankfort horizontal line were selected as the reference lines on the skull for measuring the C0-1 angle and C0-2 angle, and the consistency and repeatability were high [7, 8, 18]. Few studies have been conducted comparing reliability and reproducibility among the three measurements of upper cervical sagittal parameters. However, the ICC values of interobserver and intraobserver reliabilities for using the ML as a reference line to measure the C0-1 angle and C0-2 angle were significantly higher than those for using the FHL and FML as reference lines. The ICC values of interobserver and intraobserver reliabilities for using the FHL as a reference line to measure the C0-1 angle and C0-2 angle were slightly higher than those for using the FML as a reference line. The accuracy of measurement appears to depend on the clarity of the measurement landmarks on given radiographs. Shoda et al. found that either the basion or opisthion was difficult to identify in some of the films; the lateral bony structure of the skull base seems to render the occipital border dull and obscure [19]. We also found that the lateral bony structure of the skull base may obscure the orbitale and porion. Therefore, when we drew the McGregor line on the skull in a neutral lateral cervical radiograph, it was more stable and accurate than the MacRae line and Frankfort horizontal line. Due to the highest repeatability and consistency of the ML line as a reference line to measure upper cervical sagittal parameters, it is most advantageous to use this method for requiring stable measurement of upper cervical sagittal parameters, such as the determination of upper cervical spine sagittal parameters in upper cervical surgery and the comparison of upper cervical spine sagittal parameters before and after surgery. In addition, although using the FHL as a reference line yields a lower ICC value than using the ML when measuring the C0-1 angle and C0-2 angle, using the FHL as a reference line has a higher correlation between the C0-1 angle and C0-2 angle and the C2-7 angle than the other two lines. In the study of the compensation mechanism of sagittal alignment of the upper and lower cervical spine, use of the FHL line as the reference line for measuring the C0-1 angle and C0-2 angle is superior.

The C2-7 angle has been confirmed to be correlated with age and sex in previous studies [2, 12, 20]. Iorio et al. found that the C2-7 angle increased with aging, while the C0-2 angle did not change with age in asymptomatic people [2]. Our research also found that the correlation between the C0-1 angle and C0-2 angle measured by the three methods and the C2-7 angle gradually increased with aging. This is the result of long-term compensation of the upper and lower cervical spine to maintain horizontal visual balance. In all age groups, the correlation between the C0-1 angle and C2-7 angle of the FHL as the reference line was higher than that of the FML or ML as the reference line. The correlation between the C0-2 angle and C2-7 angle of the FHL as the reference line was also stronger than that of the FML or ML as the reference line. The correlation between the C0-2 angle and the C2-7 angle in females was higher than that in males in the study by Nojiri et al [20]. In our study, the correlation between the C0-1 angle and C2-7 angle in the female group was higher than that in the male group. In addition, the correlation between the C0-2 angle or C1-2 angle and the C2-7 angle in the female group was more advanced than that in the male group. This shows that sex is an important parameter affecting the correlation of sagittal parameters of the upper and lower cervical regions.

In some studies, the line passing through both the anterior tip of the anterior arch and the posterior tip of the posterior arch of the atlas was used as the reference line to measure the C0-1 angle and C1-2 angle [8]. However, some other studies instead take the inferior aspects of the atlas as the reference line for measuring the C0-1 angle and C1-2 angle [10, 11, 21, 22]. The C0-1 angle and C1-2 angle are often used to study the changes in cervical sagittal alignment before and after C1-2 fixation in patients with atlantoaxial dislocation [21]. Tan’s technique and Harms’ technique are commonly used in C1 screw implantation during atlantoaxial fixation [23, 24]. However, the C0-1 angle and C1-2 angle are difficult to accurately measure using the previous measurement methods because the tail of the C1 screw will obscure the middle-lower part of the posterior arch of the atlas after atlantoaxial fixation surgery by Tan’s technique or Harms’ technique. Therefore, we chose the superior aspect of the atlas as the reference line to measure the C0-1 angle and C1-2 angle. The ICC values of interobserver and intraobserver reliability of the method for measuring the C1-2 angle were 0.934 and 0.940, respectively. Pearson correlation analysis also showed that the C0-1 angle and C1-2 angle were negatively correlated with the C2-7 angle.

The present study had some limitations. First, this study was a single-center retrospective analysis, and the number of cases was small. A multicenter prospective study is required to confirm the current conclusion. Second, the three measurement methods were used and compared only in asymptomatic people in our study. However, which method is more conducive to the evaluation of cervical sagittal alignment and the study of the compensation mechanism of the upper and lower cervical spine in patients with different cervical diseases needs to be further explored.

## Conclusion

The FHL, FML, and ML all have good consistency and repeatability when used as reference lines on the skull to measure the C0-1 angle and C0-2 angle. The measurement results of the C0-1 angle and C0-2 angle with use of the three methods are all negatively correlated with that of the C2-7 angle. However, use of the ML as a reference line shows higher consistency and repeatability, and using FHL as a reference line to measure parameters of the upper cervical yields a better correlation with the C2-7 angle. The correlations between the C0-1 angle, C0-2 angle and C2-7 angle tend to increase with aging. The correlations between the C0-1 angle, C0-2 angle and C2-7 angle are stronger in females than in males. It is ideal to use the superior aspect of the atlas as the reference line to measure the C0-1 angle and C1-2 angle.

## Data Availability

Data will be available upon request to the first author, SCX.
